# Catatonia as a presentation of autism in a child: a case report

**DOI:** 10.3934/Neuroscience.2020019

**Published:** 2020-09-08

**Authors:** Robin Quilliam, Samantha Quilliam, Morag Turnbull, Shelagh Parkinson, Godwin Oligbu

**Affiliations:** Department of Paediatrics, Dr Gray's Hospital, NHS Grampian, Scotland, United Kingdom

**Keywords:** catatonia, autism, child, lorazepam

## Abstract

Catatonia commonly refers to a cluster of movement abnormalities, behaviour, volition and speech that has long been associated with psychiatric disorders in adults. Recent evidence suggests increasing prevalence in adolescents and older children with autistic spectrum disorder (ASD), but its occurrence in younger children is rare. Here we describe a 6-year-old boy presenting with catatonic autism, highlighting the diagnostic challenge and demonstrating the importance of timely assessment and management.

## 1. Introduction

Catatonia is a complex neuro-psychological disorder involving stupor, waxy flexibility and mutism lasting more than 1 hour. It has generally been regarded as an acute adult behavioural syndrome associated with schizophrenia until recently. It is believed to occur in approximately 10% of psychiatric inpatients [1]. There are increasing reports of an association between autism and catatonia [1], causing significant stress to the affected individual and their family, and resulting in a poor quality of life.

It is thought to occur as a reaction to extreme fear, interpersonal conflict, a tragic event or following significant loss [2], but the exact pathophysiology remains unknown. Neurobiologic correlates of catatonic syndromes have been identified but it is not certain if these findings constitute aetiologic causes or the sequelae of the disorder [3]. Some patients may have a genetic predisposition to catatonia and preliminary evidence suggests that genes on chromosomes 15 and 22 may be linked to the periodic form of catatonia [3].

The prevalence of catatonia in children with autism is likely to be higher than reported given the varying degree of presentation and low index of suspicion amongst clinicians. In one observational study it was proposed that catatonia may occur in 12–17% of individuals with autism spectrum disorder (ASD) [4], with the youngest patient being 15 years of age. However, little is known about catatonia in younger children with autism. Here we describe the case of a 6-year-old child with catatonia that led to diagnosis of underlying autism. Our case demonstrates the diagnostic challenges and highlights the need for a high index of suspicion in younger children.

## 2. Case presentation

A 6-year-old boy presented to our paediatric assessment unit for evaluation of persistent mutism following a hospital visit for routine venepuncture. He had been non-verbal for a few weeks and had begun using an iPad “talking board”, at which he pointed to pictures and words. Due to this persistent mutism he had stopped attending school and it became clear there had been a sudden behavioural change.

He was born at term following an uneventful pregnancy to a single mother with mental health disorder. He has two younger brothers who have no history of developmental disorder. Prior to this presentation, he maintained a weight and height above the 25^th^ centile. Apart from language delay he met his early developmental milestones. He attended nursery from age three and was assessed by age five for possible autism by the local child and adolescent mental health service (CAMHS). Although some of his behaviours appeared autistic in nature he did not meet the diagnostic threshold for ASD as he was functioning comparatively well in his first year of school.

Six months after the earlier presentation with mutism, he was found lying on the floor, still and unresponsive with no clear trigger identified. Further episodes occurred and each lasted between a few minutes and two hours. During these episodes he would not speak, move, or respond to vocal or touch stimuli. These episodes would end as abruptly as they started with him sitting bolt upright, being reactive, and crying. Subsequently, these catatonic episodes became more frequent and prolonged, lasting between ten and 48 hours. His weight dropped from the 25^th^ centile to the 0.4^th^ centile. This required admission to the local paediatric ward and a nasogastric tube (NGT) was inserted for nutrition and fluids. There was an initial difficulty in establishing a feeding regime, as he would frequently vomit. Eventually, a slow overnight feed was sufficiently tolerated. Whilst on the paediatric ward any catatonic episodes were preceded by extreme agitation, usually prior to a procedure such as passage of a NGT or venepuncture, and when ward staff attempted to restrain him he would suddenly become limp and unresponsive. He was also observed to lick and sniff ward staff that he liked and would frequently remove his NGT and offer it to staff as a gift. He became a significant flight risk with no sense of danger, requiring a wheelchair with restraining straps to attend appointments with his mother.

**Figure 1.**
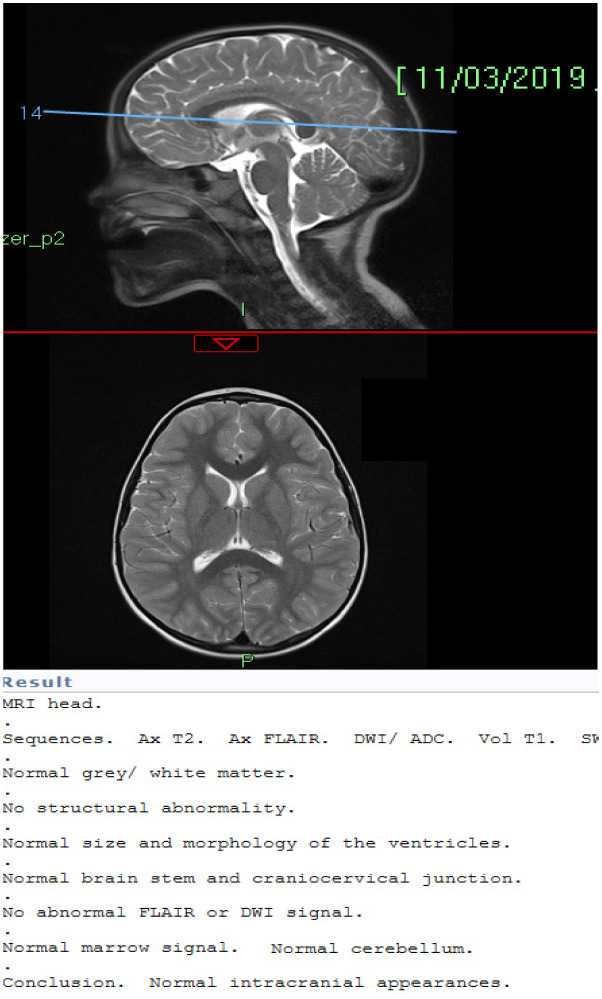
MRI brain with report.

He was assessed by the paediatric neurologist and had extensive neuro-metabolic blood investigations, an electroencephalogram (EEG), and magnetic resonance imaging (MRI) of his brain (Figure 1). These were essentially normal. He was trialled on lorazepam with a good response and an immediate reversal of his catatonia. Nevertheless, catatonic episodes continued in the absence of lorazepam and he was therefore transferred to the National Child Psychiatric Inpatient Unit (NCPIU) for five weeks for further assessment outside of his normal environment. He was commenced on regular lorazepam and his clinical condition improved significantly, with no further catatonic episodes during this admission. He was diagnosed with ASD with co-morbid catatonia because, now aged 7, he had been withdrawn from school by his mother and his social communication skills had regressed to the point he now satisfied the DSM 5 criteria for a diagnosis of ASD. Autistic symptoms (though not satisfying the DSM 5 criteria) had been present for two years prior to the development of catatonia.

On discharge from the NCIPU he struggled with transition to life at home. He developed stress-related alopecia and the frequency of catatonic episodes increased significantly. Atomoxetine was commenced following a diagnosis of co-morbid attention deficit hyperactivity disorder (ADHD). This was diagnosed on the basis of the DSM 5 criteria; he displayed a persistent pattern of inattention and hyperactivity-impulsivity that interfered with his functioning and development. Atomoxetine was soon stopped, however, due to nausea and vomiting. The lorazepam was increased to a maximum of 12 mg (0.54 mg/kg) daily which resolved his catatonic episodes. Guanfacine was commenced but soon withdrawn as the patient refused to swallow it. Methylphenidate was started and has been titrated to 15 mg twice daily which has been effective (decreasing emotional outbursts, increasing creative play and ability to self-soothe) and well tolerated with no adverse effects. Lorazepam 12 mg daily was well tolerated with no adverse effects. It has since been decreased to dose of 8 mg daily with plans to wean further.

## 3. Discussion

This case describes catatonia in a young child which led to a diagnosis of underlying ASD. Although this child was initially seen aged five for language delay and some behavioural challenges, he did not meet the DSM 5 criteria for a diagnosis of ASD because he was functioning comparatively well at school. Regression of his social communication skills following the onset of mutism aged six (and catatonia six months later) led to him being reassessed for ASD and diagnosed whilst a psychiatric inpatient. This might suggest that recent changes in diagnostic criteria, and the diagnostic procedure itself, are responsible for the under-recognition, or in this case late recognition, of ASD. Our case differs from those previously reported in terms of age of onset and the child displaying all of the hallmark characteristics of catatonia. Catatonia as a presentation of autism is rare, and to our knowledge, this is the youngest reported case of catatonia in a patient with ASD.

The child became mute aged 6 and with hindsight this was a useful indicator of the onset of catatonia. In one of the largest longitudinal studies that followed 22 patients with autism over a 12 year period, looking into the multiple faces of catatonia, 73% displayed mutism [5]. However, these patients often had many other symptoms of catatonia in addition to mutism, suggesting a low overall index of suspicion that mutism alone might predict impending catatonia in children with ASD.

Consistent with published literature, this child presented with catatonia after a trauma (routine venepuncture) on the background of a stressful home environment. Disruption of daily routine and exposure to stressful life events have been linked with the onset of catatonia in individuals with autism [5]. Due to deficits in social communication skills and heightened sensory sensitivity, studies have shown that individuals with autism may be particularly vulnerable to intense fear [6],[7]. In one adult cohort study with autism, the levels of anxiety were threefold higher than similar individuals with learning disability [6]. Catatonia was therefore postulated as an evolutionary response and coping strategy to such extreme fear or noxious stimuli in people with ASD [8]. In contrast to their findings the child in our case was not on any psychotropic medication, which has been reported as a major trigger in the development of catatonia [8]. There is evidence suggesting that a number of developmental disorders with autistic features, for example Down syndrome and Prader-Willi syndrome, can be a risk factor for developing catatonia due to general psychological and biological vulnerabilities [6],[8].

Catatonia in autism remains a diagnostic challenge. Patients presenting with features of catatonia should be assessed carefully; such assessment may require the involvement of multidisciplinary teams and thorough investigation. We undertook a full neuro-metabolic work up including genetic investigation and brain MRI. The MRI showed a normal basal ganglia (Figure 1), which has been shown to be an important structure in the pathogenesis of autism, because of its crucial role in motor and cognitive functioning [9]. Genetic testing showed no mutations on the comparative genomic hybridisation (CGH) array, particularly on genes coding for chromosomes 15 and 22, which have been linked to the periodic form of catatonia [9].

Irrespective of the underlying cause of catatonia in ASD, eliminating the trigger and prompt medical management is essential to prevent complications. This child had lost a considerable amount of weight and was dehydrated to the extent that he required a NGT for fluids and nutrition. This severity of catatonia in autism confers a risk of significant morbidity, and if not addressed in a timely fashion, may even result in fatality [10].

Benzodiazepines, especially lorazepam, remain the mainstay of treatment in children with autistic catatonia [4],[11]–[14]. Diagnosis is by means of a “lorazepam test”. If catatonia subsides, albeit temporarily, after 2 mg of lorazepam, the test is considered positive [15]. In agreement with this a significant response to 2 mg of lorazepam was seen and the child was later maintained on a higher dose to prevent further catatonic episodes. In a systematic review involving 28 individuals with autistic catatonia there was only limited evidence to support electroconvulsive therapy (ECT), lorazepam and behavioural interventions [11]. However, in patients with severe autonomic dysfunction, or life threatening symptoms as a result of catatonia, high dose lorazepam has been suggested as a suitable long term treatment [16]. If lorazepam fails, ECT is recommended [16]. Further work is needed on catatonia in autistic children, including development of any useful diagnostic and prognostic indicators.

## 4. Conclusion

This case highlights the need for a high index of suspicion of the diagnosis of autism in children who present with features of catatonia. Our case report is unique in its recognition of catatonia in such a young patient, and subsequent treatment of the catatonia with high dose lorazepam. We propose that assessment for ASD should be considered in children presenting with catatonia even if typical autistic features have not previously been evident. The diagnosis of ASD may prove difficult in the presence of mutism or catatonia. However, delays in the diagnosis of ASD may complicate management of catatonia and be associated with a poorer outcome. If catatonia is diagnosed, early intervention should be implemented before stupor, which is more difficult to manage, occurs.
